# Simulation of English Speech Recognition Based on Improved Extreme Random Forest Classification

**DOI:** 10.1155/2022/1948159

**Published:** 2022-07-01

**Authors:** Chunhui Hao, Yuan Li

**Affiliations:** ^1^English Department, Shijiazhuang Tiedao University, Shijiazhuang, Hebei, China; ^2^Public Educational Department, Xingtai Medical College, Xingtai, Hebei, China

## Abstract

Existing speech recognition systems are only for mainstream audio types; there is little research on language types; the system is subject to relatively large restrictions; and the recognition rate is not high. Therefore, how to use an efficient classifier to make a speech recognition system with a high recognition rate is one of the current research focuses. Based on the idea of machine learning, this study combines the computational random forest classification method to improve the algorithm and builds an English speech recognition model based on machine learning. Moreover, this study uses a lightweight model and its improved model to recognize speech signals and directly performs adaptive wavelet threshold shrinkage and denoising on the generated time-frequency images. In addition, this study uses the EI strong classifier to replace the softmax of the lightweight AlexNet model, which further improves the recognition accuracy under a low signal-to-noise ratio. Finally, this study designs experiments to verify the model effect. The research results show that the effect of the model constructed in this study is good.

## 1. Introduction

In recent years, speech recognition technology has made great developments and is quickly applied to the product field, and language recognition systems have sprung up like mushrooms [1]. Transliteration is usually used for the translation of named entities, such as place names and names, which refers to the use of the similarities and differences in the pronunciation rules of the source language and the target language to translate the source language form into the target language form [2]. Since transliteration deals with translation problems from the perspective of pronunciation and has good results in dealing with the translation of unregistered words, it has a wide range of applications in many cross-language tasks such as machine translation and bilingual maps. Among them, bilingual maps have very high requirements for transliteration, and strict transliteration rules must be followed to obtain bilingual annotations that can be used. Currently, there are a large number of transliteration rules available, and the China Geographical Names Committee has formulated a list of transliteration rules in 50 languages. These rule tables are divided into vowels and consonants according to the international phonetic symbols corresponding to the different pronunciations of the letters of the original language after romanization, and they are pronounced by vowels and consonants alone or in combination. Research on transliteration is generally divided into two categories: research on transliteration equivalent pair mining and research on transliteration model construction. The former refers to mining dual transliteration equivalent pairs from parallel or comparable corpora to construct a larger and newer transliteration dictionary. The latter refers to the use of parallel bilingual corpus for training to automatically build a transliteration model based on its own information and contextual information [3].

Speech recognition is an all-encompassing subject, which involves many different fields, including linguistics, acoustics, statistics, and artificial intelligence, and is also called automatic speech recognition [4]. Its goal is to display the vocabulary content in the human voice on the computer as computer-readable information, which can be recognized by the computer. If speech recognition technology is combined with other natural language processing technologies, more complex but convenient applications can be constructed, such as combining machine translation and speech synthesis to obtain speech translation [5]. Due to the importance and great advantages of speech recognition in all aspects, many companies invest a lot of manpower and material resources in research.

With the improvement of the level of technology, the development of the internet has brought huge amounts of language and text information, and the amount of data in web page text is also increasing day by day. At the same time, the phenomenon of multiple languages has gradually appeared. The language recognition system can hand over a large amount of repetitive and tedious labor to the machine to handle, saving manpower and material resources, and improving work efficiency [6]. The research of language recognition focuses on the improvement and optimization of algorithms and models, and its main methods are through linguistics and machine learning methods. In addition, methods of probability statistics or information theory have also been widely adopted, and speech recognition methods have been successfully applied in practice [7].

## 2. Related Work

There are also many other methods used in short text language recognition. The literature described a method of using affix tables to expand the dictionary and used a parallel corpus to test it [8]. The literature proposed a method combining the n-gram language model with the naive Bayes classifier to achieve the purpose of classifying language types [9]. The literature proposed a method to realize language recognition based on the user's previous messages and the related content embedded in the messages for Twitter information [10]. This method not only uses the information of the word itself but also effectively uses the information between words, which greatly improves the efficiency of language recognition of short texts. During this period, many language recognition tools were developed. As deep learning technology becomes more and more mature, many researchers have begun to think about how to apply deep learning technology to language recognition and have made many attempts. However, through practice, it can be found that deep learning technology has good effects in the field of speech. At the same time, language recognition technology for the field of speech has become more and more mature. However, for short texts, as the corpus continues to improve, machine learning methods based on statistics have become simpler and more efficient. In addition, the traditional n-gram-based language recognition has a strong dependence on the dataset. The literature pointed out that achieving good recognition results on six European corpora does not mean that the same good results will be achieved on corpora containing more languages [11]. The literature pointed out in the evaluation experiment of each language recognition model that the accuracy of the same model on different datasets is also very different. At the same time, it also pointed out that removing noise in the dataset, such as special characters in the Twitter dataset, has obvious help to improve the recognition rate [12].

For different audio types, different audio features are used. The literature used two acoustic characteristics of zero-crossing rate and short-term energy to classify voice and music in broadcast signals [13]. The literature first divided the audio signal in the TV into mute, signal with music component and signal without music component by using four audio characteristics of short-term energy, zero-crossing rate, pitch frequency, and spectral peak trajectory [14]. Then, it further divided the signal containing music component into pure music, singing voice, and voice with music background, and further divided the signal without music component into pure audio and noisy audio. The literature used five characteristics: MFCC, zero-crossing rate, short-term energy, spectral centroid, and spectral width, and introduced three characteristics of spectral differential amplitude, sub-band period, and the proportion of noise frame to divide the audio signal into 5 categories: pure speech, impure speech, music, environmental sound, and silence [15].

## 3. Modulated Signal Model

The modulation methods of the modulation signal mainly include single-frequency modulation, linear frequency modulation, nonlinear frequency modulation, frequency encoding, phase encoding, and frequency-phase encoding hybrid modulation. Due to different types of voice waveforms, in order to meet their performance requirements, different modulation types are selected, and low-intercept probability waveform modulation methods are usually used. Moreover, this waveform is characterized by low transmit power and low interception. Its original simple single-frequency continuous-wave modulation has been continuously developed to multiple frequency modulation, linear frequency modulation, nonlinear frequency modulation, phase code modulation, and other more complex modulation technologies. Therefore, different modulation methods are used for different types of voice waveforms, thereby reflecting various modulation signal characteristics. Therefore, this study studies the following 10 common modulated signal models to prepare for the subsequent identification of various modulated signals.

### 3.1. Normal Signal (NS)

Normal signal (NS) FM signal is the most basic signal form in the modulation signal. Because of its simple transceiver structure and lower transmit power, it can get a larger signal-to-noise ratio than other modulated signals, and it has good detection performance and low-interception performance. Therefore, conventional voice signals are widely used in various voice systems. Its mathematical expression is shown by the following formula. Among them, *A* is the amplitude, *f*_0_ is the carrier frequency, *φ* is the initial phase, and *T* is the pulse width.

(1) $$\begin{array}{c} s\left(t\right)=\left\{\begin{array}{cc} A\;\exp\left\{j\left(2\pi f_{0}t+\varphi \right)\right\}, & 0\leq t\leq T,\\ 0, & \text{else.} \end{array}\right. \end{array}$$

Binary phase-shift keying (BPSK) modulated signal has good antinoise and low-intercept performance characteristics. Therefore, it is widely used in various communication systems and voice communication systems. Its mathematical expression is shown by the following formula. Among them, *M* is the number of phases. When *M*=2 is a two-phase coded BPSK signal, the value of *ϕ*_*i*_ is 0 and *π* in two phases, *N* is the number of symbols, and *T*_*p*_ is the width of the symbols. The BPSK phase encoding can be realized by Barker code, Frank code, etc. Moreover, the characteristic of the BPSK signal is that its instantaneous frequency undergoes a phase jump at the code element change of the code sequence.

(2) $$\begin{array}{c} s\left(t\right)=A\sum_{i=1}^{N}\exp\left\{j\left(2\pi f_{0}t+\varphi \right)\right\}u_{T_{p}}\left(t-iT_{p}\right),\\ \phi_{i}\in \left\{\frac{2\pi }{M}\left(m-1\right),\;m=1,2,\text{…},M\right\}. \end{array}$$

Linear frequency modulation (LFM) modulation signal is the most typical modulation method in frequency modulation. Because of its large time-bandwidth product, low transmit power, high-range resolution, and low-intercept probability, it is widely used in various high-performance voices and sonars. Its mathematical expression is as follows. Among them, A is the frequency modulation slope, and *B* is the frequency modulation bandwidth.

(3) $$\begin{array}{c} s\left(t\right)=\left\{\begin{array}{cc} A\;\exp\left\{j\left(2\pi f_{0}t+\frac{1}{2}\pi kt^{2}+\varphi \right)\right\}, & 0\leq t\leq T,\\ 0, & \text{else.} \end{array}\right. \end{array}$$

Nonlinear frequency modulation (NLFM) modulation signals are also commonly used modulation signals for frequency modulation. Because the LFM signal passes through the matched filter and the output waveform has a high sidelobe, it easily leads to low-range resolution and loss of the detected target. However, the NLFM signal can obtain lower sidelobes and has a larger time-bandwidth product, which can improve the measurement accuracy and distance resolution of the voice system. Its mathematical expression is very similar to the LFM signal, except that the power of the highest polynomial of the phase is greater than 2. Its mathematical expression is shown by the following formula. Among them, *k*_1_, *k*_2_, *φ* are the modulation coefficients and the initial phase of the signal, respectively.

(4) $$\begin{array}{c} s\left(t\right)=\left\{\begin{array}{cc} A\;\exp\left\{j\left(\begin{array}{c} 2\pi f_{0}t+\frac{1}{2}\pi k_{1}t^{2}\\ +\frac{1}{2}\pi k_{2}t^{3}+\varphi \end{array}\right)\right\}, & 0\leq t\leq T,\\ 0, & \text{else.} \end{array}\right. \end{array}$$

The frequency-hopping Costas modulated signal has good antifading, antinoise, and low-intercept performance characteristics, and it is widely used in frequency-hopping communication. Its realization is to divide the signal whose pulse width is *T* into *N* equal parts of subpulses. Among them, the length of the subpulse is *T*_*s*_=*T*/*N*, and the time sequence of each frequency hopping is {*T*_1_, *T*_2_,…, *T*_*N*_}. According to the given Costas coding sequence {*θ*_1_, *θ*_2_,…, *θ*_*N*_}, the frequency sequence {*f*_1_, *f*_2_,…, *f*_*N*_} in each period of time can be obtained. The frequency selection in each time interval should satisfy the following:

(5) $$\begin{array}{c} f_{k+i}-f_{k}\neq f_{i+j}-f_{j},\\ 1\leq k<i+j\leq \theta_{N}. \end{array}$$

Among them, *p*_*n*_ is the subpulse. When the subpulse *p*_*n*_ is a single-frequency conventional signal, the frequency of the subpulse in each time interval is *f*_*i*_=*θ*_*i*_/*T*_*s*_=*θ*_*i*_Δ*f*. Among them, *i* ∈ {1,2, ⋯, *N*}, and Δ*f* is the frequency step amplitude.

(6) $$\begin{array}{c} s\left(t\right)=\sum_{n=0}^{N-1}p_{n}\left(t-nT_{s}\right),\\ p_{n}=\left\{\begin{array}{cc} A\;\exp\left\{j\left(2\pi f_{m}t+\varphi \right)\right\}, & 0\leq t\leq T_{s},\\ 0, & \text{else.} \end{array}\right. \end{array}$$

The emergence of Frank polyphase encoding is to improve the problem of higher sidelobe peaks in FM signals after matched filters and Doppler sensitivity in phase-encoded signals. Therefore, it is often widely used in various voice communication systems. When the signal pulse width is *T*, its phase change is realized by *M* frequency steps. Among them, each frequency step has *M* sets of sampling points, so its total length *N*_*c*_=*M*^2^. At the same time, the phase change of the Frank code whose total length is *N*_*c*_ is represented by the *M* × *M* matrix. Among them, the phase change of the *M* sampling points of the *M* group takes 0 as the initial value and is incremented by (*M* − 1)(2*π*/*M*).

(7) $$\begin{array}{c} s\left(t\right)=\left\{\begin{array}{cc} A\;\exp\left\{j\left(2\pi f_{0}t+\phi_{ij}\right)\right\}, & 0\leq t\leq T,\\ 0, & \text{else}, \end{array}\right.\\ \phi_{ij}=\frac{2\pi }{M}\left(i-1\right)\left(j-1\right),\\ i=1,2,\text{…},M;j=1,2,\text{…},M,\\ \frac{2\pi }{M}\left[\begin{array}{ccccc} 0 & 0 & 0 & \cdots & 0\\ 0 & 1 & 2 & \cdots & M-1\\ 0 & 2 & 4 & \cdots & 2\left(M-1\right)\\ \vdots & \vdots & \vdots & \vdots & \vdots \\ 0 & M-1 & 2\left(M-1\right) & \cdots & {\left(M-1\right)}^{2} \end{array}\right]. \end{array}$$

### 3.2. Multitime Coded Signals (*T*1 and *T*4)

Multitime code *T*1(*n*),2(*n*), *T*3(*n*), *T*4(*n*) is a code sequence composed of different time periods given to each of their phase states. Among them, *T*1(*n*) and *T*2(*n*) are generated based on the step frequency, and *T*3(*n*), *T*4(*n*) is generated based on the approximate LFM signal waveform.

**Figure 1:**
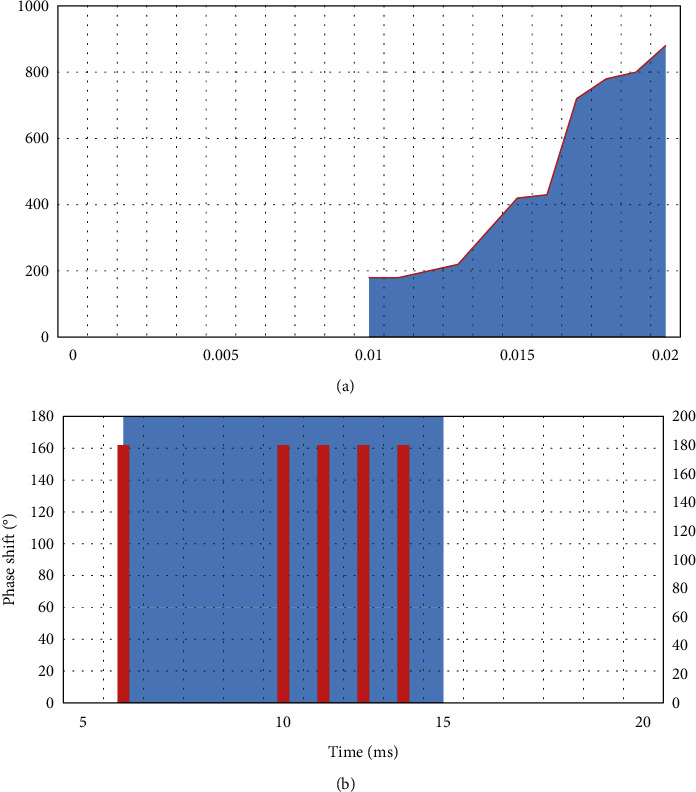
*T*1(*n*) multitime coding. (a) *T*1(2) code with unprocessed phase change. (b) *T*1(2) code after phase change processing.

**Figure 2:**
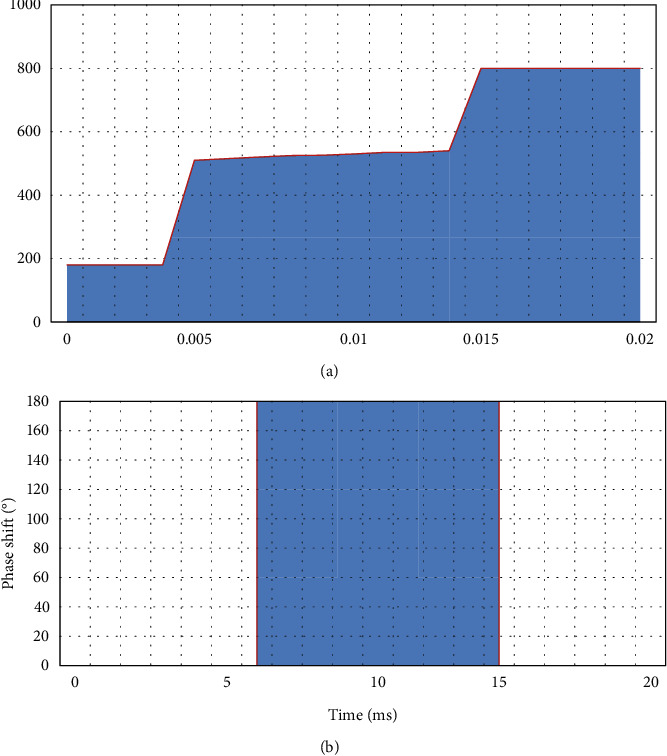
*T*2(2) multitime coding. (a) *T*2(2) code with unprocessed phase change. (b) *T*2(2) code with unprocessed phase change.

**Figure 3:**
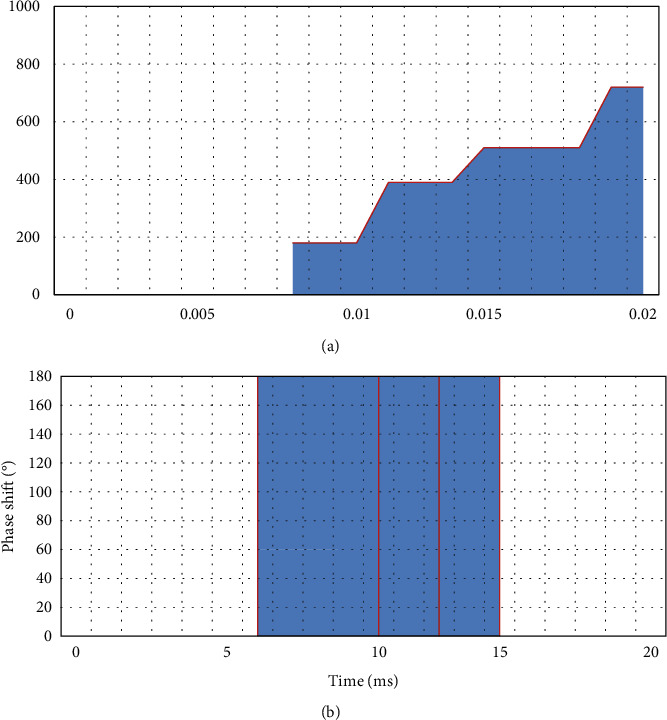
*T*3(2) multitime coding. (a) *T*3(2) code with unprocessed phase change. (b) *T*3(2) code after phase change processing.

(1) The mathematical expression of phase change of *T*1(*n*) multitime code sequence is as follows:
  (8) $$\begin{array}{c} \phi_{T1\left(t\right)}=\mathrm{mod}\left\{\frac{2\pi }{n}INT\left[\left(kt-jT\right)\frac{jn}{T}\right],2\pi \right\}. \end{array}$$
Among them, *n* is the number of phase states of the *T*1(*n*) sequence, *j*=0,  1,2,…, *k* − 1 is the step-frequency segment number of the multitime code *T*1(*n*) sequence, and *k* is the number of segments of the *T*1(*n*) code sequence. At the same time, *t* is the time, and *T* is the duration of the entire multitime encoding.
The *T*1(*n*) coding sequence is analyzed for phase-shift characteristics, as shown in Figure 1. The phase state, number of segments, and encoding time of the *T*1(*n*) encoding sequence are, respectively, selected as *n*=2, *k*=3, *T*=20*ms* to obtain the unprocessed phase change in Figure 1(a) and the processed phase change in Figure 1(b).
(2) The meaning of the mathematical expression parameters of the phase change of the *T*2(*n*) multitime code sequence is the same as the above *T*1(*n*) code.
  (9) $$\begin{array}{c} \phi_{T2\left(t\right)}=\mathrm{mod}\left\{\frac{2\pi }{n}\text{INT}\left[\left(kt-jT\right)\left(\frac{2j-k+1}{T}\frac{n}{2}\right)\right],2\pi \right\}. \end{array}$$
Similarly, the *T*2(*n*) code sequence is analyzed for phase-shift characteristics, as shown in Figure 2. The phase state, number of segments, and encoding time of the *T*2(*n*) encoding sequence are, respectively, selected as *n*=2, *k*=3, *T*=18*ms*, and the unprocessed phase change in Figure 2(a) and the processed phase change in Figure 2(b) are obtained.
(3) The mathematical expression of the phase change of the *T*3(*n*) multitime code sequence is shown by the following formula. Among them, *n* is the number of phase states, Δ*F* is the modulation bandwidth, and *t*_*m*_ is the modulation period.
  (10) $$\begin{array}{c} \phi_{T3\left(t\right)}=\mathrm{mod}\left\{\frac{2\pi }{n}\text{INT}\left[\frac{n\Delta Ft^{2}}{2t_{m}}\right],2\pi \right\}. \end{array}$$
Similarly, *T*3(*n*) code sequence bei1 performs phase-shift characteristic analysis, as shown in Figure 3. The signal-to-noise ratio, bandwidth, phase status, number of segments, and coding time of the *T*3(*n*) coding sequence are, respectively, selected as 2  *dB*  , Δ*F*=250*Hz*, *n*=2, *k*=3, *t*_*m*_=18*ms*, and the unprocessed phase change in Figure 3(a) and the processed phase change in Figure 3(b) are obtained.
(4) The meaning of the parameters in the mathematical expression of the code phase change of *T*4(*n*) for a long time is the same as that of *T*3(*n*) as follows:
  (11) $$\begin{array}{c} \phi_{T4\left(t\right)}=\mathrm{mod}\left\{\frac{2\pi }{n}INT\left[\frac{n\Delta Ft^{2}}{2t_{m}}-\frac{n\Delta Ft}{2}\right],2\pi \right\}. \end{array}$$

**Figure 4:**
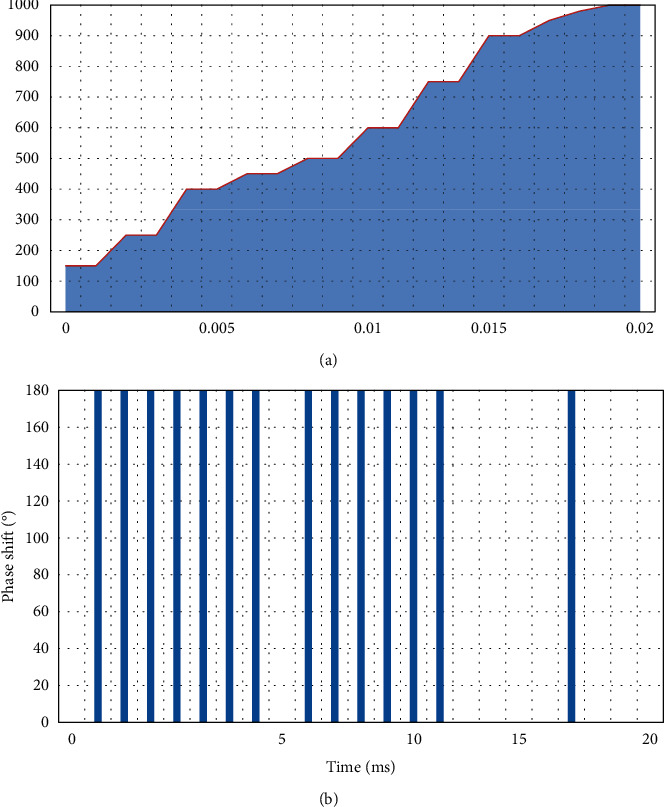
*T*4(2) multitime coding. (a) *T*4(2) code with unprocessed phase change. (b) *T*4(2) code after phase change processing.

As shown in Figure 4, the characteristics of the *T*4(*n*) coding sequence are analyzed. The signal-to-noise ratio, bandwidth, phase status, number of segments, and coding time of the *T*4(*n*) coding sequence are selected as 2 dB, Δ*F*=250 Hz, *n*=2, *k*=3, *t*_*m*_=18 ms, respectively, the unprocessed phase change is shown in Figure 4(a), and the processed phase change is obtained in Figure 4(b).

### 3.3. Time-Frequency Analysis Method Analysis

There are many classic time-frequency analysis methods, such as short-time Fourier transform (STFT), wavelet transform (WT), Wigner–Ville transform, Choi–Williams distribution (CWD), and Hilbert Huang transform (HHT) transformation, and so on. These methods mainly consist of linear and nonlinear transformations. The linear time-frequency transformation satisfies the linear superposition principle, while the nonlinear transformation does not satisfy the superposition principle, and it will introduce cross terms, thereby affecting the correctness of the signal's time-frequency characteristics. Among them, the Wigner–Ville transform and Choi–Williams transform belong to the Cohen time-frequency distribution. As shown by the following formula, Cohen-like time-frequency analysis gives a general mathematical representation. When *g*(*η*, *τ*) takes different kernel functions, it can correspond to different types of bilinear (quadratic) time-frequency division transforms.

(12) $$\begin{array}{c} C\left(t,\Omega :g\right)=\frac{1}{2\pi }\int \int \int x\left(u+\frac{\tau }{2}\right)x^{*}\left(u-\frac{\tau }{2}\right),\\ g\left(\eta ,\tau \right)e^{-j\left(\eta t+\Omega \tau -u\eta \right)}\text{d}u\text{d}\tau \text{d}\eta . \end{array}$$

The STFT is a linear transformation in time-frequency analysis. It was first used by Gabor, so it is also called the Gabor transform. Because the actual signal is an infinitely long nonstationary signal, it does not satisfy the Fourier transform (FT) condition. However, the STFT uses a window function to divide an infinitely long signal into several finite-length “small segments.” Each “small segment” is approximately stationary so as to satisfy the Fourier transform conditions. The set obtained by the Fourier transform of each “small segment” is the fast Fourier transform. The mathematical expression of STFT is as follows:

(13) $$\begin{array}{c} {\text{STFT}}_{x}\left(t,\Omega \right)=\int_{-\infty }^{\infty }x\left(\tau \right)g^{*}\left(\tau -t\right)e^{-j\Omega \tau }\\ =\langle x\left(\tau \right),g\left(\tau \right)e^{j\Omega \tau }\rangle . \end{array}$$

Compared with the Fourier transform, the advantage of the short-time Fourier transform is that it can obtain the instantaneous frequency information of the signal at different times by increasing the window function. The physical meaning is clear and clear, and the STFT is a linear transformation. Unlike other nonlinear time-frequency analyses, it has no interference terms, so the time-frequency resolution is high.

However, the shortcomings of the short-time Fourier change are also obvious because it has a window function. Therefore, its time-frequency resolution is limited by Heisenberg's uncertainty principle (Δ*t*Δ*f* ≥ 1/4*π*), and the real-time frequency resolution cannot be high at the same time. Moreover, once the STFT determines the window length of the window function, the corresponding time-frequency resolution is also determined. Therefore, STFT is a single-resolution analysis method. When the analyzed signal contains multiple frequency components, the short-time Fourier transform method cannot satisfy the time-frequency analysis of multiple frequency components.

The CWD time-frequency analysis is a nonlinear time-frequency analysis method. In the Cohen time-frequency analysis, when the kernel function *g*(*η*, *τ*)=e^−*η*^2^*τ*^2^/*σ*^ can get the CWD time-frequency analysis, the mathematical expression of the CWD time-frequency analysis is shown by the following formula:

(14) $$\begin{array}{c} CWD\left(t,f\right)=\int \int A_{x}\left(\eta ,\tau \right)g\left(\eta ,\tau \right)\exp\left(-j2\pi \left(\eta t-tf\right)\right)\text{d}\eta \text{d}\tau ,\\ A_{x}\left(\eta ,\tau \right)=\int \int x\left(t+\frac{\tau }{2}\right)x^{*}\left(t-\frac{\tau }{2}\right)\exp\;{\left(-j2\pi t\eta \right)}^{-j2\pi \tau }\text{d}t,\\ g\left(\eta ,\tau \right)=\exp\left[-\sigma {\left(\eta \tau \right)}^{2}\right]. \end{array}$$

In the above formulas, *g*(*η*, *τ*) is the kernel function, *σ* is the controllable parameter factor, and CWD suppresses the cross term mainly by changing the *σ* parameter factor. If the value of *σ* is large, the CWD time-frequency analysis becomes the WVD time-frequency analysis. Although it can improve the time-frequency aggregation of signal terms, it will cause serious cross-term interference. If the value of *σ* is small, although the interference of the cross term is small, the amplitude and frequency of the signal change very slowly, which is easy to form a tail, resulting in poor time-frequency aggregation of the signal. Therefore, in order to balance the resolution of the signal and the cross-term interference, *σ* needs to select an appropriate value.

The CWD distribution needs to select a suitable kernel function and a suitable controllable factor to effectively suppress the cross-term interference, and then obtain a time-frequency image with a higher time-frequency resolution. However, the implementation of CWD is more complicated, and proper kernel function and parameter size need to be selected. When the signal contains high-frequency components, the selected parameter factors cannot separately reflect the time-frequency characteristics of the high and low frequencies of the signal. Therefore, at this time, the frequency aggregation and the ability to suppress the cross term are not as good as the smooth pseudo-Wigner–Ville distribution.

The Wigner–Ville time-frequency analysis is a nonlinear time-frequency transformation. When the kernel function *g*(*η*, *τ*)=1 is Cohen's time-frequency distribution, the WVD time-frequency distribution is obtained. Since it does not involve a window function, it is not restricted by Heisenberg's uncertainty principle (Δ*t*Δ*f* ≥ 1/4*π*) and, therefore, has a higher time-frequency resolution. However, it will produce serious cross-term interference when performing time-frequency analysis on multiple signals. Its mathematical expression is as follows:

(15) $$\begin{array}{c} WVD_{x}\left(t,\Omega \right)=\int_{-\infty }^{\infty }x\left(t+\frac{\tau }{2}\right)x^{*}\left(t-\frac{\tau }{2}\right)e^{-j\Omega \tau /2}\text{d}\tau . \end{array}$$

Since the WVD time-frequency analysis method is a nonlinear transformation, as shown by the following formula, when there are multiple signals *x*(*t*)=*x*_1_(*t*)+*x*_2_(*t*) undergoing WVD time-frequency transformation, the superposition theorem will not be satisfied. The WVD distribution of the sum of multiple signals is not equal to the sum of the WVD distribution of each signal, so cross-term interference will occur.

(16) WVDxt,Ω=∫−∞∞x1t+τ2+x2t+τ2x1∗t−τ2+x2∗t−τ2,e−jΩτ/2dτ=Wx1,x1t,Ω+Wx2,x2t,Ω+2ReWx1,x2t,Ω.

Among them, the above formulas *W*_*x*_1_,*x*1_(*t*, Ω) and *W*_*x*_2_,*x*2_(*t*, Ω) are the self-time-frequency distributions of *x*_1_(*t*) and *x*_2_(*t*) (referred to as signal terms). 2Re[*W*_*x*_1_,*x*2_(*t*, Ω)] is the mutual time-frequency distribution of *x*_1_(*t*) and *x*_2_(*t*) (referred to as the cross term), which is the interference generated when multiple signals are analyzed in WVD frequency. These cross terms weaken the energy of the signal term in the time-frequency domain, causing aliasing and blurring of the time-frequency image. Therefore, in order to obtain a higher time-frequency resolution, the interference of the cross term in WVD should be suppressed, and the energy of the signal term should be enhanced.

When the pseudo-Wigner–Ville distribution (pseudo-WVD, PWVD) appears, in order to eliminate the influence of cross-term interference, it is implemented by smoothing the WVD windowing function *h*(*τ*). Its mathematical expression is as follows:

(17) $$\begin{array}{c} {\text{PWVD}}_{x}\left(t,\Omega \right)=\int_{-\infty }^{\infty }x\left(t+\frac{\tau }{2}\right)x^{*}\left(t-\frac{\tau }{2}\right)h\left(\tau \right)e^{-j\Omega \tau /2}\text{d}\tau . \end{array}$$

In the formula, *h*(*τ*) is a window function. The shorter the window length in the time domain, the more obvious the smoothing effect in the frequency domain, and the better the effect of eliminating cross terms. The result of PWVD windowing makes the complete nonlocality of WVD become localized, which greatly improves the signal analysis performance, makes the edges smoother, and has fewer miscellaneous items. However, it reduces the frequency resolution. Therefore, it eliminates the cross term at the cost of reducing the resolution, which is not the best time-frequency analysis method.

### 3.4. Smooth Pseudo-Wigner–Ville Transform

Smooth pseudo-WVD (SPWVD) adds a window function *g*(*u*) based on PWVD to smooth the time domain, which further reduces the interference of cross terms. The two window functions of SPWVD are equivalent to two simple two-dimensional low-pass filters, which can smooth the time domain and frequency domain by selecting an appropriate window length. Its mathematical expression is as follows:

(18) $$\begin{array}{c} {\text{SPWVD}}_{x}\left(t,\Omega \right)=\int_{-\infty }^{+\infty }\int_{-\infty }^{+\infty }x\left(t-u+\frac{\tau }{2}\right)x^{*}\left(t-u+\frac{\tau }{2}\right)h\left(\tau \right)g\left(u\right)e^{\frac{-j\Omega \tau }{2}}\text{d}\tau \text{d}u. \end{array}$$

In the above formula, because the window functions *h*(*τ*) and *g*(*u*) are smoothed in both frequency and time domains, it has the best effect of suppressing the cross term. At the same time, it can maintain a good compromise in the choice of time-frequency resolution and energy concentration, so that time-frequency images have both good time-frequency resolution and better energy concentration. Therefore, in all Cohen-type time-frequency analyses, the SPWVD time-frequency analysis has a good ability to suppress the signal cross term, and the degree of removal of the cross term can be controlled by the window function. Therefore, choosing SPWVD time-frequency analysis can obtain a higher time-frequency resolution.

**Figure 5:**
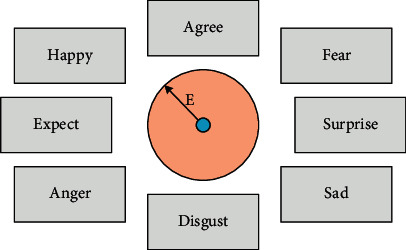
Emotion wheel.

With the rapid development of information technology and the increasing dependence of human beings on computers, researchers have paid more and more attention to human-computer interaction capabilities. How to improve this ability? To improve, it must be able to perceive the surrounding environment and atmosphere, and the attitude and emotion of the object. In this way, it is possible to provide the most comfortable dialogue environment for the dialogue object, to eliminate the obstacles between the operator and the machine. At present, the research on emotional information processing has entered the critical stage, and the research on emotional information processing in speech signals has received the most attention. The emotional information contained in the speech signal is a very important information resource. It is an essential part of the information for people to perceive things. In real life, different people speak differently, and the same person will also show different emotional factors at different times. The information conveyed was only one word short, but it was completely different. In the beginning, voice processing did not take these points into consideration, and the technical level at the time did not support this difference. Therefore, these emotions that are difficult to express with algorithms are virtually omitted. With the development of technology, this is obviously not acceptable to the public. What means to identify these subtle gaps will need to be sought in future research to improve the level of speech emotion recognition. The emotion research of speech signals needs to make an effective and reasonable classification of speech emotion according to certain characteristic standards. Then, the properties of the characteristic parameters are studied based on different categories. After years of research, we believe that emotions are distributed on a circular structure, and the center of the structure is the natural origin. Relative to the natural origin, the points distributed on the circular structure are the state of various emotional factors. The strength of these emotional factors on the circle is too weak to be reflected. It expands in different directions through the circumference, and each direction is an emotional manifestation. Emotion has intensity, and it spreads outward from the natural origin. The more outside the circle, the greater the intensity, and the size of emotion is calculated according to a certain proportion. We call this kind of emotions arranged in a circle around the natural origin as the “emotion wheel.” As shown in Figure 5, for each sentence we input with emotion, we can find a point in the emotional circle that matches the intensity of the emotion. Connecting this point with the origin is the direction the emotion points to, and the only vector obtained is the emotion sentence that is different from other emotions. In this two-dimensional plane, the direction of the vector is the meaning represented by the emotion, and the magnitude of the vector is the intensity of the emotion.

## 4. Model Effect Analysis

**Figure 6:**
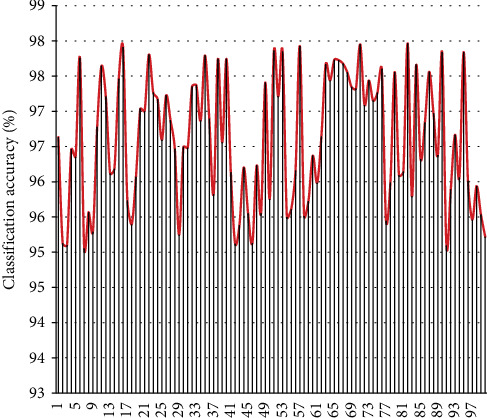
Statistical diagram of voice classification effect.

After constructing the above English and speech recognition model, the performance of the model is analyzed. This study analyzes the pronunciation of 100 English majors as examples. First, this study analyzes the effect of the model English speech classification, and the results are shown in Figure 6.

**Figure 7:**
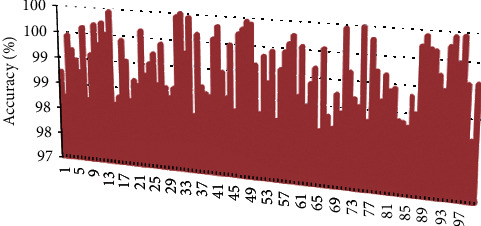
Statistical diagram of the accuracy of English speech recognition.

From the above statistical effects of speech classification, it can be seen that the model constructed in this study has a certain effect. On this basis, the accuracy of English speech recognition is verified. This study takes the English pronunciation of 100 English majors as an example to analyze, and each person reads 100 English sentences and uses this system for speech recognition. The results obtained are shown in Figure 7.

As shown in Figure 7, the accuracy of the model constructed in this study is as high as 98% for English speech recognition, which shows that the effect of the model constructed in this study is good.

## 5. Conclusions

With human demand for speech products, as a technical difficulty in speech signal processing, the process of marketization of speech synthesis is accelerating. In order to adapt to the market and apply voice products to all aspects of the market, in addition to improving the quality of synthesized speech and enhancing the expressiveness of speech synthesis, it also needs to be satisfied in terms of practicability. This study uses deep learning algorithms to perform time-frequency analysis on the massive data collected to obtain signal time-frequency images for training and extract the time-frequency characteristics of the signal from training to obtain the internal distribution law of the data. Moreover, this study establishes the best probability model to classify and identify massive data. This method finds the internal distribution of data through the training of massive data, and it can not only accurately identify the signal in the case of signal parameter distortion, signal loss, etc., but also train various voice waveforms at the same time. After the model is constructed, the performance of the model is verified and analyzed, and the analysis is carried out from the perspectives of language classification and speech recognition. The results obtained show that the model constructed in this study is effective and can be applied in practice.

## Data Availability

The data used to support the findings of this study are available from the corresponding author upon request.
